# Brain perfusion asymmetry in patients with oral somatic delusions

**DOI:** 10.1007/s00406-013-0390-7

**Published:** 2013-01-29

**Authors:** Yojiro Umezaki, Ayano Katagiri, Motoko Watanabe, Miho Takenoshita, Tomomi Sakuma, Emi Sako, Yusuke Sato, Akira Toriihara, Akihito Uezato, Hitoshi Shibuya, Toru Nishikawa, Haruhiko Motomura, Akira Toyofuku

**Affiliations:** 1 Psychosomatic Dentistry, Graduate School of Medical and Dental Sciences, Tokyo Medical and Dental University, 1-5-45 Yushima, Bunkyo-ku, Tokyo, 113-8549 Japan; 2 Psychosomatic Dentistry Clinic, Dental Hospital, Tokyo Medical and Dental University, Tokyo, Japan; 3 Complete Denture Prosthodontics, Graduate School of Medical and Dental Sciences, Tokyo Medical and Dental University, Tokyo, Japan; 4 Department of Diagnostic Radiology and Oncology, Graduate School of Medical and Dental Sciences, Tokyo Medical and Dental University, Tokyo, Japan; 5 Department of Psychiatry and Behavioral Sciences, Graduate School of Medical and Dental Sciences, Tokyo Medical and Dental University, Tokyo, Japan

**Keywords:** Oral cenesthopathy, Delusional disorder somatic type, SPECT, Brain perfusion asymmetry

## Abstract

Oral cenesthopathy is a somatic delusion or hallucination involving the oral area and is categorized as a delusional disorder, somatic type. The pathophysiology of this intractable condition remains obscure. In this study, we clarified the pathophysiology of oral cenesthopathy by evaluating regional brain perfusion. We performed single photon emission computed tomography (SPECT) using ^99m^Tc-ethylcysteinate dimer in 16 subjects (cenesthopathy:control = 8:8). The SPECT images were visually assessed qualitatively, and quantitative analyses were also performed using a three-dimensional stereotactic region-of-interest template. The visual assessment revealed a right > left perfusion asymmetry in broad areas of the brain among the patients. The quantitative analysis confirmed that the regional cerebral blood flow values on the right side were significantly larger than those on the left side for most areas of the brain in the patients. A comparison of the R/(R + L) ratios in both groups confirmed the significant brain perfusion asymmetry between the two sides in the callosomarginal, precentral, and temporal regions in the patients. Qualitative evaluation of the SPECT images revealed right > left brain perfusion asymmetry in broad regions of the brain. Moreover, the quantitative analyses confirmed the perfusion asymmetry between the two sides in the frontal and temporal areas. Those may provide the key for elucidation of the pathophysiology of oral cenesthopathy.

## Introduction

Oral cenesthopathy is a somatic delusion or hallucination involving the oral area and is categorized as a delusional disorder, somatic type (DDST). Patients complain of unusual sensations without corresponding abnormal findings in the oral area, such as excessive mucus secretion, a slimy sensation in the mouth, or a feeling of coils or wires being present within the mouth.

Because of their firm conviction that their annoying symptoms have a somatic base, patients often visit dental clinics, without consulting a psychiatrist. Most of these patients are reluctant to see a psychiatrist, even if they are recommended to consult one. To make matters worse, the symptoms in most cases are drug-resistant, resulting in dentist shopping.

Previous case reports examining the regional cerebral blood flow (rCBF) of patients with DDST have demonstrated hypoperfusion in the left temporal and left parietal lobes [10, 24, 27, 34]; this hypoperfusion was successfully reversed by pharmacological treatment or modified electroconvulsive therapy (mECT). We also previously reported a case of oral cenesthopathy in which a rightward asymmetry of blood flow in the temporal area of the brain disappeared after mECT [33].

Unlike the relatively consistent results described in case reports, statistical SPECT studies analyzing multiple DDST patients are very few and have reported inconsistent results. Tateno et al. [32] reported that patients with oral cenesthopathy exhibited hyperperfusion in the thalamus and right anterior cingulate, as compared to patients with depression. Another recent study examining DDST patients reported hyperperfusion in the left post-central gyrus and right paracentral lobule, which are regarded as the primary sensory areas [25].

Although some studies have examined the relationship between functional brain images and DDST, most of these studies have been case reports. Moreover, to the best of our knowledge, the qualitative and quantitative differences between the rCBF in patients with oral cenesthopathy and normal control subjects have never previously been analyzed. The purpose of this study was to clarify the pathophysiology of oral cenesthopathy through qualitative and quantitative evaluations of brain perfusion using ^99m^Tc-ECD SPECT.

## Methods

### Patients with oral cenesthopathy

The subjects were outpatients of the Department of Psychosomatic Dentistry, Tokyo Medical and Dental University, Tokyo, Japan. Diagnosis of oral cenesthopathy had been made based on the chief complaints of the subjects of experiencing abnormal oral sensations. The patients’ complaints were limited to the oral area. All of the patients were able to maintain their social standard of living, although they were somewhat troubled by bizarre sensations. There were no other psychiatric symptoms, and a psychiatrist confirmed the absence of either depression or schizophrenia. No cognitive deficits were detected, and the measured revised Hasegawa dementia scale (HDS-R) scores of the patients were >25. No abnormalities were observed on magnetic resonance image (MRI) of the brain, except for a high signal intensity on the T2-weighted images that was not significant.

The demographic data of the patients are listed in Table 1. The patients consisted of 2 men and 6 women ranging in age from 66 to 83 years (75.9 ± 6.0 years). The duration of the symptoms ranged from 10 to 72 months (29.8 ± 20.4 months). All patients were right-handed as defined by the Edinburgh inventory [26].

**Table 1:** Demographic data for patients with oral cenesthopathy

Case no.	Age	Sex	Handedness	Duration since onset (months)	Occupation	Complaints at first examination	Side of symptom (left or right)	Delusional conviction	Neuropathic symptoms	Diagnosis by psychiatrist or physician
1	80	M	Right	15	A former public servant	“Feels the presence of wires in the mandibular incisors when removing dentures”	Both sides	−	Nothing	Neurosis
2	70	F	Right	72	A former textile designer	“Feels slimy saliva”, “feels like her teeth are made of iron and is sore from chewing”	Both sides	+	Moving like eating something	Somatoform disorder
3	80	F	Right	10	A homemaker	“Feels like some fluid is flowing from her mandible”	Both sides	−	Nothing	Nothing significant
4	73	F	Right	44	A homemaker	“Feels like bubbles are flowing behind her dentures”	Both sides	−	Nothing	Nothing significant
5	66	M	Right	35	A former advertising agent	“Feels like gas is blowing up in his mouth”, “feels like something is struggling, as if there is an animal in his mouth”	Both sides	+	Chewing	Delusional disorder
6	74	F	Right	21	A homemaker	“Feels something sticky coming up rapidly in her mouth”, “feels like a membrane is covering and squeezing her incisors”	Both sides	−	Nothing	Nothing significant
7	83	F	Right	25	A homemaker	“Feels like trash is coming up behind her dentures”, “feels sliminess in her mouth”	Both sides	−	Nothing	Oral dysesthesia
8	81	F	Right	16	A homemaker	“Feels sliminess in her mouth”, “feels that her lips are dry”	Both sides	−	Sticking out her tongue	Cenesthopathy

Delusional conviction: delusional conviction for bizarre sensation as an alien substance

SPECT was performed after obtaining verbal consent from the patients. Written informed consent was obtained from all the patients for this study.

### Control subjects

Eight age- and sex-matched subjects (75.8 ± 5.8 years, 2 men and 6 women) without symptoms of oral cenesthopathy were enrolled as the control subjects. The inclusion criteria were (1) referral for brain perfusion SPECT imaging for dementia screening, (2) no neurological or psychiatric symptoms, (3) HDS-R score >25, and (4) no detectable abnormalities visible on MRI examination of the brain, except for a high signal intensity on T2-weighted images that was not significant. All data were collected from the subjects’ medical records. The handedness of the control subjects was not identified.

This study was conducted with the approval of the Ethical Committee of Tokyo Medical and Dental University.

### Brain perfusion SPECT

The subjects were requested to remain in a comfortable supine position with their eyes closed in a quiet room. Data acquisition was begun after a bolus injection of 600 MBq of ^99m^Tc-ECD via the right brachial vein. First, the passage from the heart to the brain was monitored using a rectangular large-field, dual-head gamma camera (E.CAM Signature; Toshiba, Tokyo, Japan) equipped with low-energy, high-resolution, parallel-hole collimators. Data acquisition consisted of a sequence of 100 frames at a rate of 1 s/frame using a 128 × 128 matrix. Five minutes after the injection of ^99m^Tc-ECD, SPECT images were obtained using the same gamma camera equipped with fan-beam collimators. The energy window was set at 140 keV ± 15 %, and 45 step-and-shoot images were obtained throughout 180 degrees of rotation (128 × 128 matrix, 1.72 mm/pixel) with an acquisition time of 30 s/step. All the images were reconstructed using ‘ordered subsets expectation maximization’ (OSEM) and then smoothed three dimensionally using a Butterworth filter. The reconstructed images were corrected for gamma ray attenuation using the Chang method.

To quantify the rCBF, the Patlak plot method [20, 21] was applied to the ^99m^Tc-ECD cerebral blood perfusion SPECT images to measure the mean CBF. Quantitative flow-mapping images were then obtained from the qualitative cerebral perfusion SPECT images using the Patlak plot graphical analysis and Lassen’s correction [7, 19].

### Data analysis

#### Visual assessment

A physician specialized in the field of nuclear medicine who was unaware of the patient grouping evaluated all the qualitative SPECT images of all the 16 study subjects. The interpreter visually decided whether or not any asymmetry in brain perfusion existed in each of the four cerebral lobes (frontal, temporal, parietal, and occipital), lenticular nucleus, thalamus, and cerebellum.

#### Quantitative analysis

The rCBF quantification was performed using a program called “3DSRT”, an abbreviation for three-dimensional stereotaxic regions of interest (ROI) template [16, 31]. 3DSRT is a fully automated rCBF quantification program that can be used for examining a total of 636 ROIs. These 636 ROIs are categorized into 12 brain segments on the 3DSRT template: callosomarginal, precentral, central, parietal, angular, temporal, posterior cerebral, pericallosal, lenticular nucleus, thalamus, hippocampal, and cerebellar segments. The blood flow to each ROI was quantified in mL/100 g/min.

For the quantitative analysis, the rCBF values obtained from the 3DSRT data were used.

As an index of brain perfusion asymmetry, the right to right + left ratio [R/(R + L) ratio] was calculated for the 12 brain segments in each subject as follows:

R/(R + L) ratio = rCBF values for the concerned segment on the right side × 100/sum of the rCBF values for the corresponding segments on the right and left sides.

The mean rCBF values and mean R/(R + L) ratios for each brain segment were calculated separately for the patients and the control subjects. The results are expressed as mean ± standard deviation (SD).

### Statistical analysis

The Mann–Whitney *U* test was performed using the PASW 17.0 software (IBM, Chicago, IL, USA). *P* values of <0.05 were considered to indicate statistical significance; all tests were two-tailed.

## Results

### Clinical features of the patients with oral cenesthopathy in this study

Table 1 lists the clinical features of the eight patients with oral cenesthopathy enrolled in this study. The patient group was composed mainly of elderly women. The sex and age distributions, shown in this table, were consistent with those in our previous paper [33]. The patients’ chief complaints (for example, sticky saliva or bubbles flowing behind their dentures or wires for the mandibular incisors when removing dentures) were strange, bizarre, and hard to empathize with. Moreover, the symptoms were persistent, remaining unchanged over long periods of time, which led to persistent complaints from the patients. Despite the extraordinary complaints, most of the patients who came to our clinic seemed to be polite and well-adjusted socially and to have no remarkable personality problems. Most of the patients were aware that their distressing symptoms were very strange and rarely acceptable to others. Their unusual symptoms were localized to the oral area and did not affect any other body areas. None of the patients were diagnosed as having schizophrenia, depression, or dementia (HDS-R >25) by a psychiatrist who examined them.

### Visual qualitative assessment of the SPECT images

**Fig. 1:**
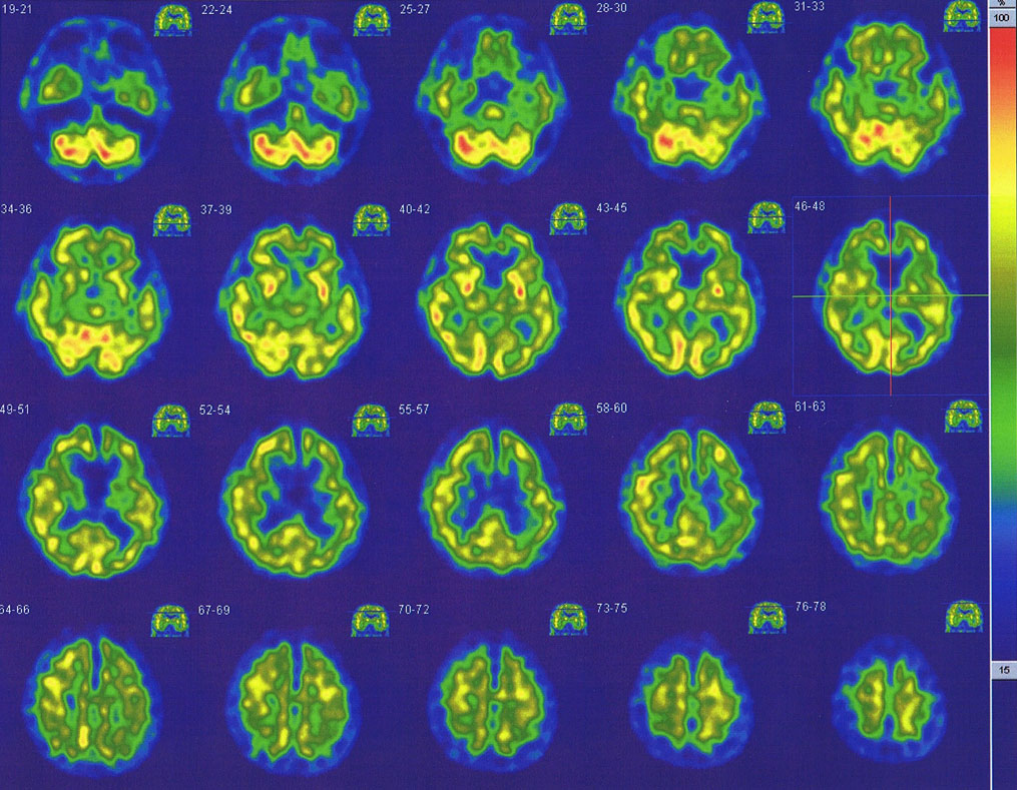
^99m^Tc-ECD SPECT images of a patient with oral cenesthopathy (case no. 1 in Table 1). As compared with the values on the left side, the right temporal lobe and right frontal lobe show higher perfusion values

Figure 1 shows representative SPECT images obtained according to the above-mentioned methods (case no. 1 in Table 1). Visual assessments of the images in the 8 control subjects revealed a right > left perfusion asymmetry (R > L) in 8 brain areas in total (frontal in 2, temporal in 1, parietal in 1, lenticular nucleus in 2, and thalamus in 2 cases) and a left > right perfusion asymmetry (L > R) in 4 brain areas in total (occipital in 1, lenticular nucleus in 1, and thalamus in 2 cases). In the 8 patients, on the other hand, R > L and L > R perfusion asymmetries were observed in 17 brain areas (frontal in 4, temporal in 4, lenticular nucleus in 3, thalamus in 4, and cerebellum in 2 cases) and 4 brain areas (lenticular nucleus in 1 and cerebellum in 3 cases), respectively. Thus, R > L perfusion asymmetry seemed to be more frequent among the patients than among the control subjects, especially in the frontal and temporal areas and the thalamus.

### Quantitative analysis

Table 2 shows the mean rCBF values for each segment for the control subjects and the patients, as obtained using 3DSRT. In the control subjects, significant differences in the rCBF values between the right and left side were observed only in the cerebellar area. In the patients, on the other hand, significant differences of the rCBF were distributed over broad areas of the brain, including the parietal, temporal, thalamus, lenticular nucleus, callosomarginal, and angular segments. Thus, the characteristic right > left blood flow asymmetries in broad areas of the brain in the patients were confirmed by the 3DSRT analyses.

**Table 2:** The mean cerebral blood flow values

Segment	Right	Left	*P* value
Control subject			
Callosomarginal	42.15 ± 4.23	41.95 ± 4.14	0.528
Precentral	44.47 ± 4.51	43.81 ± 3.90	0.123
Central	43.09 ± 6.54	43.65 ± 6.10	0.183
Parietal	42.43 ± 5.08	42.23 ± 6.22	0.327
Angular	44.51 ± 6.46	44.24 ± 5.81	0.944
Temporal	41.52 ± 4.43	41.03 ± 3.43	0.889
Posterior cerebral	46.52 ± 4.93	46.13 ± 5.20	0.208
Pericallosal	44.17 ± 4.92	43.66 ± 4.99	0.050
Lenticular nucleus	48.40 ± 6.15	48.13 ± 5.96	0.674
Thalamus	48.16 ± 8.31	47.26 ± 6.17	0.575
Hippocampus	33.94 ± 3.76	33.89 ± 3.06	0.779
Cerebellum	51.70 ± 4.53	50.38 ± 4.52	0.017*
Oral cenesthopathy			
Callosomarginal	43.11 ± 6.20	41.33 ± 6.50	0.025*
Precentral	46.04 ± 6.55	44.20 ± 6.28	0.069
Central	45.08 ± 5.37	44.29 ± 5.33	0.208
Parietal	44.59 ± 7.19	42.80 ± 6.25	0.012*
Angular	48.81 ± 8.38	46.49 ± 7.45	0.036*
Temporal	43.75 ± 5.07	41.15 ± 4.56	0.012*
Posterior cerebral	49.11 ± 6.32	47.84 ± 5.96	0.069
Pericallosal	44.59 ± 7.11	43.45 ± 7.21	0.093
Lenticular nucleus	51.82 ± 6.07	49.90 ± 5.98	0.017*
Thalamus	47.64 ± 5.55	44.53 ± 5.99	0.012*
Hippocampus	37.56 ± 5.02	37.07 ± 4.98	0.674
Cerebellum	58.14 ± 6.83	57.13 ± 7.32	0.161

*rCBF* regional cerebral blood flow

* *P* < 0.05

**Fig. 2:**
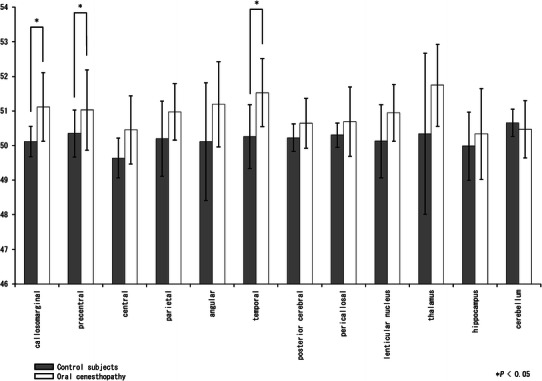
‘R/(R + L) ratios’ for each brain area. A Mann–Whitney *U* test was used to compare the ‘R/(R + L) ratios’ for various areas of the brain between the control subjects and the patients. The *X*-axis shows each region, and the *Y*-axis shows the ‘R/(R + L) ratio’. The graphs on the left (*gray bars*) and the right (*white bars*) show the mean ± SD for the control subjects and the patients, respectively. The ROIs with a significant difference of the ratio are denoted by a single asterisk (*P* < 0.05)

Figure 2 shows the ‘R/(R + L) ratio’ for each of the segments in the control subjects and the patients with oral cenesthopathy, as calculated using the above-mentioned method. We observed significantly larger ‘R/(R + L) ratios’ in the callosomarginal, precentral, and temporal segments in the patients, which also confirmed the R > L blood flow asymmetries in the callosomarginal, precentral, and temporal segments of the patients’ brains.

## Discussion

The major findings of this study were as follows: (1) right > left blood flow asymmetry was found in broad areas of the brain in the patients with oral cenesthopathy, whereas no such perfusion asymmetries were found in the control subjects, in either the visual or 3DSRT analyses; and (2) calculation of the ‘R/(R + L) ratio’, which is used to quantify rCBF asymmetries, revealed significantly larger asymmetries in the callosomarginal, precentral, and temporal regions of the brain in the patients, as compared with the differences in the control subjects.

Detailed examination of the results revealed, however, that the results shown in Table 2 and Fig. 2 were similar, but not identical. For example, a remarkably significant difference of the blood flow in the thalamus is seen in Table 2 but not in Fig. 2. This discrepancy between the results illustrated in Table 2 and Fig. 2 is attributable, in part, to the large variances in the patients and control subjects. Among the various brain areas, however, the callosomarginal and temporal areas seemed to exhibit consistent right > left blood flow asymmetry in both analyses.

To determine whether the right > left rCBF asymmetry in broad areas of the brain was due to absolute hypoperfusion on the left side or due to hyperperfusion on the right (opposite) side, or both the differences in the rCBF between the patients and control subjects were analyzed by Statistical Parametric Mapping, version 5 (SPM5; Wellcome Department of Imaging Neuroscience, University College, London, United Kingdom). This analysis revealed no regions with significant differences (data not shown).

To the best of our knowledge, the only prior SPECT study to statistically analyze the rCBF in a reasonable number of oral cenesthopathy patients was the one that compared the rCBF between patients with oral cenesthopathy and those with depression. According to this previous report, patients with oral cenesthopathy showed significantly higher rCBF values in the right anterior cingulate and bilateral thalamus than the patients with depression [32]. Although a direct comparison of this study with our present study is difficult, the finding of right > left blood flow asymmetry in the anterior cingulate, which is regarded as being part of the callosomarginal area, on 3DSRT, is interesting.

In regard to asymmetries in cerebral blood flow, many studies have been conducted in healthy volunteers [3, 28]. In a recent study, Brinkmann et al. [1] reported left > right perfusion asymmetry in widespread areas of the cerebral hemispheres in SPECT images of neurologically normal volunteers, based on examination of the images using semi-automated ROI analysis and statistical parametric mapping (SPM). In view of this previous report, our present results showing right > left rCBF asymmetry in patients with oral cenesthopathy could have important implications.

Numerous studies have reported functional asymmetries of the rCBF between the two hemispheres in patients with pathological mental states [8, 13, 15, 18]. Using SPECT or positron emission tomography (PET) imaging, pathological perfusion asymmetries, especially in the frontal and temporal areas, have been documented in numerous studies, although the results have been inconsistent. For example, in patients with schizophrenia, unilateral (usually on the left side) or bilateral temporal lobe hypoperfusion [22, 29, 35] frequently associated with frontal lobe hypoperfusion [14, 22, 35], has been reported. In patients with depression, decreased or increased rCBF has been observed in the frontal area of one or both sides, occasionally accompanied by left temporal hypoperfusion [5, 17]. Similarly, right > left perfusion asymmetry has been observed in patients with post-traumatic stress disorder, childhood autism, and others [2, 30].

Focusing on the relationships between perfusion asymmetry and clinical symptoms, some previous studies have demonstrated attenuation of the perfusion laterality with improvement of the mental condition after treatment [4, 9].

In terms of the DDST, some interesting rCBF studies have shown improvement of the decreased rCBF in the left temporal and parietal regions after successful treatment [10, 24, 34] or modified electroconvulsive therapy (mECT) [27]. Recently, we also reported a patient with oral cenesthopathy in whom hyperperfusion in the right relative to the left temporal lobe improved after mECT, in parallel with alleviation of the clinical symptoms [33]. Irrespective of whether right-side hyperperfusion or left-side hypoperfusion exists, a predominant asymmetry in the right side compared with the other side of the rCBF that is attenuated by subsequent successful treatment may be a common characteristic of these previous reports. Our result suggesting a right-side-predominant asymmetry is consistent with these previous reports.

On the other hand, Nemoto et al. [25] reported that patients with DDST exhibited a significant increase of perfusion in the left post-central gyrus and right paracentral lobule, both of which are involved in somatic sensory processing. As discussed in their paper, their patients’ primary symptoms might have been somatic hallucinations that subsequently developed into somatic delusions, suggesting that various types of DDST may have been included in the same category.

Indeed, although the patients with DDST in this study were clinically selected (socially well-adjusted people whose delusions or hallucinations were limited to the oral area), the asymmetrical perfusion patterns did not seem to be homogeneous, suggesting that various subgroups may have again been included in this clinical entity. Further investigations are needed for a precise interpretation.

The most interesting question in this study was why the right > left brain perfusion asymmetry was observed in such broad areas of the brain. A straightforward speculation is that the characteristic CBF distribution might be a consequence of abnormalities in the thalamus or basal ganglia, such as the lenticular nucleus, which are closely connected to several cortices. In a previous paper focusing on delusional infestation (DI), a type of DDST, Freudenmann et al. [6] hypothesized that DI might be caused by dopamine transporter dysfunction in the striatum, influencing the fronto-striato-thalamo-parietal network (also see [11, 12]). Our speculation that abnormalities in the thalamus or basal ganglia may underlie the rCBF asymmetry seen in patients with oral cenesthopathy is partially similar to the aforementioned hypothesis in relation to the pathophysiology of DI.

There were two limitations to the present study. The first was that all the patients were receiving small amounts of various medications, such as antidepressants, antipsychotic agents, etc. Since it has been reported that antidepressants [17] and antipsychotics [4, 23] could affect the rCBF, the asymmetrical patterns observed in this study could have been influenced to some degree by medications that have a minimal effect on the clinical symptoms. The other was that there were only 8 patients and 8 control subjects. Because of this small number of subjects, it was difficult to detect statistically significant differences with adequate dependability. For more reliable study of the brain SPECT findings in oral cenesthopathy, a greater number of subjects are needed.

In conclusion, this study was the first to carry out qualitative as well as quantitative analysis of the differences in the rCBF between patients with oral cenesthopathy and control subjects. Right > left blood flow asymmetries were found in broad areas of the brain in the patients with oral cenesthopathy, and the ‘R/(R + L) ratios’ in the frontal and temporal brain areas were significantly higher in the patients as compared to the values in the control subjects.

Further studies are needed to clarify the pathophysiology of oral cenesthopathy in greater detail and to develop safe treatments with favorable outcomes.
